# Concordance between practitioner questionnaire responses and observed clinical treatment recommendations for treatment of dentin hypersensitivity: findings from the National Dental Practice-Based Research Network

**DOI:** 10.1186/s12903-019-0772-y

**Published:** 2019-06-14

**Authors:** Mark S. Litaker, Dorota T. Kopycka-Kedzierawski, D. Brad Rindal, Jeffrey L. Fellows, Marc W. Heft, Cyril Meyerowitz, Sidney Chonowski, Gregg H. Gilbert, The National Dental PBRN Collaborative Group

**Affiliations:** 1 Department of Clinical and Community Sciences, University of Alabama at Birmingham, 1919 7th Avenue South, Birmingham, AL 35294-0007 USA; 2 Eastman Institute for Oral Health, University of Rochester, 625 Elmwood Ave, Rochester, NY 14620 USA; 3 Health Partners Institute for Education and Research, 8170 33rd Avenue South, Mail Stop 21111R, PO Box 1524, Bloomington, MN 55440-1524 USA; 4 Kaiser Permanente Center for Health Research, 3800 N Interstate Avenue, Portland, OR 97227 USA; 5 Department of Oral & Maxillofacial Surgery, College of Dentistry, University of Florida, Gainesville, FL 32610-0416 USA; 6 Private Practice of General Dentistry, 66 Maple Ave, Morristown, NJ 07960 USA

**Keywords:** Dentin hypersensitivity, National Dental Practice-Based Research Network, Agreement, Treatment modalities

## Abstract

**Background:**

Few published reports have presented concordance between treatment choices selected by dentists in hypothetical clinical scenarios and treatment choices made by the same dentists in actual clinical practice. The aim of the current cross-sectional study, conducted within the Management of Dental Hypersensitivity (MDH) study, was to assess the potential value of practitioners’ questionnaire responses regarding their typical treatment provided for management of dentin hypersensitivity (DH), by evaluating agreement between these responses and subsequently-observed recommendations recorded during actual clinical examinations.

**Methods:**

A total of 171 practitioners enrolled in the National Dental Practice-Based Research Network completed both a questionnaire and a clinical study regarding methods they use to treat dental hypersensitivity. The questionnaire solicited first-, second- and third-choice products when prescribing or recommending management of dentin hypersensitivity. Agreement was calculated for first-choice products/recommendations and for inclusion in the top three choices, as identified by the practitioners, from 11 listed treatment options. Overall percent agreement and Cohen’s kappa statistic were calculated, with associated 95% confidence intervals (CI). Associations between practitioner characteristics and agreement were also evaluated.

**Results:**

For individual treatment modalities, percentage agreement ranged from 63 to 99%, depending on the specific item. Percentage agreement between typical treatment and actual treatment for each practitioner’s top three treatment modalities, as a combined grouping, ranged from 61 to 100%. When these same agreement pairings were quantified to account for agreement above that expected by chance, kappa values were poor to low.

**Conclusions:**

Concordance between hypothetical clinical scenarios and treatment choices made by the same dentists in actual clinical practice showed moderate to high levels of percentage agreement, but Cohen’s kappa values suggested relatively low levels of agreement beyond that expected by chance. This analysis adds to the larger work of the network which has now observed a wide range of agreement between hypothetical and actual care, depending upon the specific diagnosis or treatment under consideration.

Questionnaire data for DH might serve as a useful adjunct to clinical data regarding treatment recommendations, but agreement was not sufficiently high to justify use of questionnaires alone to characterize patterns of treatment for this particular condition.

**Electronic supplementary material:**

The online version of this article (10.1186/s12903-019-0772-y) contains supplementary material, which is available to authorized users.

## Background

Previous studies have evaluated concordance between dentists’ pre- and post-treatment observations in clinical practice [1], between dentists’ questionnaire responses to hypothetical scenarios and responses collected during clinical treatments occurring subsequently [2, 3], between different hypothetical scenarios [4], and between dentists’ questionnaire responses and previously-published clinical standards of care [5, 6].

For example, one study calculated agreement between estimated depths of primary caries lesions, before and after operative treatment, for 8351 lesions in actual clinical practice. Unadjusted percentage agreement between dentists’ pre- and post-operative depth assessments was 69% overall, 88% for lesions that were into the inner one-third of dentin and 52% for lesions in the outer one-half of enamel [1].

Other studies have reported concordance based on questionnaires or hypothetical scenarios alone, with no direct observation of clinical practice [4–7]. Few published reports have presented concordance between treatment choices selected by dentists in hypothetical clinical scenarios and treatment choices made by the same dentists in actual clinical practice. Heaven, et al., evaluated the association between dentists’ decision to repair or to replace existing restorations in three hypothetical scenarios with treatment provided to patients in comparable clinical circumstances [3]. Percent agreement was not estimated, but dentists who recommended repair rather than replacement in more of the hypothetical scenarios did indeed have higher rates of repair in clinical practice. In a different study, pre-treatment estimates of lesion depths at which dentists placed initial restorations during a clinical study were compared with lesion depths at which they stated that they would place restorations in hypothetical scenarios [2]. The primary interest was in estimating the percentage of restorations for which the actual caries depths were less than the threshold depths indicated in the hypothetical scenarios.

The current analysis was conducted as a part of a National Dental Practice-Based Research Network study, the Management of Dentin Hypersensitivity (MDH) Study. The objective of the MDH study was to evaluate the methods used by dentists to diagnose and treat dentin hypersensitivity (DH) [8, 9]. The National Dental Practice-Based Research Network (the Network) is a consortium of dental practices and dental organizations focused on improving the scientific basis for clinical decision-making and moving evidence into everyday clinical practice. The structure and function of the Network have been published previously [10], and detailed information about the network is available online [11].

The objective of the current analysis was to evaluate concordance between stated preferences in treatment recommendations for DH, based on practitioners’ responses to a pre-study questionnaire, with observed recommendations made by the same practitioners during subsequent actual clinical treatment of their patients who were enrolled in the MDH study, which served as the parent study for this analysis. The purpose of evaluating concordance was to aid in assessing the value of practitioners’ responses to questions regarding their typical practice methods, as these might be more easily obtained than observations based on actual patient visits.

## Methods

### The MDH study

Any general dentist or specialist who was enrolled in the Network at the full participation level was eligible to enroll in the study. Study practitioners completed all necessary human participant and conflict-of-interest training as required by the network.

Detailed procedures and inclusion and exclusion criteria for the MDH study have been previously reported [8, 9]. Briefly, the study was reviewed and approved by the institutional review board of the home institution of the lead investigator (D.K.K.), as well as by those for each of the 6 administrative regions within the network. After enrolling in the study, practitioners completed an online questionnaire before enrolling patients. The results of the pre-study questionnaire have been previously published [8].

The practitioners recruited dental patients from their respective practices who reported having sensitive teeth and subsequently received a diagnosis of DH. Each patient provided informed consent to participate in the study. Patient inclusion criteria included that the patient was willing to be available for the duration of the study; was 19 years old or older; reported having a sensitive tooth or teeth with a diagnosis of DH (third molars were excluded), and that the patient granted permission for follow-up [8, 9]. The exclusion criteria for patients were having a medical condition that could interfere with reliable pain reporting, having a chronic pain condition, having odontogenic pain, or reported having taken analgesics more than 3 times in the past week.

At the baseline visit, the practitioners completed the patient’s oral examination, confirmed the diagnosis of DH, completed a dental history form, and recommended, prescribed, or applied treatment for DH. Patients completed pain assessment forms at the baseline visit and at 1-, 4-, and 8-weeks post-visit. Sample size considerations were based on precision of estimation of percentages, adjusted for the effect of clustered sampling due to enrollment of multiple patients per dental practice. On the basis of this analysis, the target sample size of the study was set at approximately 180 practitioners and 1900 patients.

### The agreement study

The current study is a cross-sectional analysis of agreement between: (1) practitioners’ characterization of their typical treatments for DH as specified on the MDH study pre-study questionnaire; and (2) summary rates of treatment selections made during the baseline examination visit, calculated from each practitioner’s sample of patients enrolled in the MDH study.

### Practitioner and practice data: the enrollment questionnaire

Practitioners completed a Network enrollment questionnaire that collected information about themselves and their practices. The questionnaire is publicly available online [12]. Selected practitioner and practice characteristics were used to explore potential associations with agreement between predicted (typical) and observed treatment recommendations.

### Treatment recommendations, predicted: the practitioner questionnaire

The initial stage of practitioner participation in the MDH study comprised completing an online questionnaire regarding methods used to diagnose and manage or treat DH, as well as the practitioner’s judgment regarding factors that predispose a patient to DH. The network’s applicable Institutional Review Boards approved the study. All participants provided informed consent after receiving a full explanation of the procedures.

Development and testing of the questionnaire have been previously reported [8]. Briefly, the questionnaire was pilot tested using six dental practitioners who evaluated the length, acceptability and internet browser compatibility. Following revision based on this initial review, test-retest reliability of the questionnaire was evaluated for 24 additional practitioners [8].

Respondents were asked to select all procedures they routinely use to manage DH from the following list: (1) Fluoride products (includes gels, varnishes pastes and rinses), (2) Desensitizing OTC potassium nitrate toothpastes, (3) Glutaraldehyde/HEMA products, (4) Bonding agents, (5) Sealants, (6) Restorative treatments, (7) Lasers, (8) Oxalates, (9) No treatment, (10) Advice, and (11) Other. These are termed “treatment modalities” on the questionnaire. “No treatment” principally included dentists’ advice and counseling regarding modifying toothbrushing rigor and type of toothbrushes or toothpaste type, reduction in the ingestion of acidic foods and drinks or discontinuing the use of tooth-whitening products.

Following the selection of individual treatments, the practitioners were asked to rank their first, second and third choices of the items they had selected. Results of the questionnaire have been previously reported [8]; the questionnaire is publicly available online [13].

### Treatment recommendations, observed: the patient examination form

On patient examination forms, practitioners selected “treatment recommended and/or prescribed” from a provided list and were instructed to check all applicable items. The list items were the same as those on the Questionnaire, except that there were separate items for five fluoride products. For analysis, these were collapsed into a single “fluoride products” category in order to correspond to the single questionnaire item for these products.

For each dentist, frequencies were tabulated for each treatment recommendation. Percentages of the dentists’ total recommendations were calculated for each recommendation. These percentages were ranked for each dentist to identify the most common and the top three recommendations. If two treatment recommendations were tied for most frequently used, then both of these were coded as the first choice for that dentist. These would be evaluated separately as agreement or disagreement with the questionnaire responses.

The patient examination form is publicly available online [14]. The practitioner questionnaire, baseline patient examination form and STROBE checklist for the Agreement Study are included as Additional files 1, 2 and 3.

### Statistical analysis

Data analysis was conducted using SAS® Release 9.4 software.

Frequencies of each product/treatment recommendation were tabulated for the patients who were examined by each practitioner, and these were ranked to identify the practitioner’s first, second and third most frequently recommended treatment modalities. Dichotomous variables were coded to identify whether each item was the first-choice treatment recommendation and whether each item occurred among the top three most-used recommendations for each dentist.

Agreement was calculated between (1) dentists’ typical product/treatment recommendations (based on the pre-study questionnaire) with (2) the observed rankings based on these same dentists’ actual treatment recommendation (as recorded during their patient examinations). Agreement was evaluated separately for each listed product/recommendation, for the product/recommendation indicated as the dentist’s first-choice and for products/recommendations indicated as within the dentist’s top three recommendations. Unadjusted percentage of agreement was used to characterize concordance between survey (typical) and (actual) clinical usage. Cohen’s kappa statistic was used to evaluate agreement beyond that expected by chance. Point estimates and 95% confidence intervals were calculated for each measure.

Secondary analyses were conducted to evaluate practitioner and practice characteristics as potential predictors of agreement on the top three treatment recommendations. The outcome variables for these analyses were variables representing agreement between survey responses and clinical use for each treatment recommendation occurring among the three most common choices. The practitioner/practice characteristics that were evaluated as potential predictors were practitioner gender, race (categorized as white versus other due to small observed numbers of non-white respondents), age group (27–39, 40–49, 50–59, 60–73), full-time versus part-time, practice location (Inner city of urban area; Urban, not inner city; Suburban; Rural), and number of practice locations. Associations between practitioner/practice characteristics and agreement were evaluated using the chi-square statistic, separately for each treatment recommendation. No adjustment for multiple analyses was used.

## Results

### Practices/practitioners and patients

A total of 185 dentists from all six network regions completed the practitioner questionnaire. Of these, 171 completed patient clinical examination forms for a total of 1862 patients and were included in the current study. Characteristics of the practitioners and patients have been previously reported [8, 9]. Patient ages ranged from 19 to 87 (median = 45).

The median number of patients enrolled per practitioner was 12, with enrollment ranging from 1 to 16 patients. Six practitioners (3.5%) enrolled a single patient each, and 21 practitioners (12.3%) enrolled three or fewer. Sixty six practitioners (38.6%) enrolled 16 patients each.

Regional distribution of the practitioners was relatively uniform, with 16.4–17.0% of the total from each of the Midwest, Southwest, South Central and South Atlantic regions. 14.6% were from the Western region and 18.1% were from the Northeast region.

### Agreement of survey responses with clinical recommendations

Percent agreement and Cohen’s kappa statistic, representing agreement beyond that expected by chance, are presented in Table 1 for first-choice treatment recommendations. Desensitizing OTC potassium nitrate toothpaste was the product that was most often selected by the dentists as their first choice recommendation, with 84 dentists (49.1%) choosing this item. Of these, 49 dentists (58.3%) also indicated this recommendation most often in the clinical examinations. Overall agreement on this recommendation was 63.7% (95% CI: 56.1, 70.9), with kappa = 0.27 (95% CI: 0.13, 0.42).

**Table 1:** Agreement on first-choice treatment recommendation for management of dentin hypersensitivity

Treatment Recommended	Product Listed as First Choice on Survey	Agreement Between Survey and Exam	Kappa
	n (%)	% (95% CI)	(95% CI)
Desensitizing OTC Potassium Nitrate Toothpaste	84 (49.1%)	63.7% (56.1, 70.9)	0.27 (0.13, 0.42)
Fluoride Products	62 (36.3%)	63.2% (55.5, 70.4)	0.22 (0.07, 0.37)
Glutaraldehyde/HEMA Products	6 (3.5%)	95.9% (91.8, 98.3)	0.20 (−0.16, 0.57)
Advice	6 (3.5%)	93.0% (88.1, 96.3)	− 0.04 (−0.06, − 0.02)
Other	6 (3.5%)	88.9% (83.2, 93.2)	0.13 (−0.08, .034)
Restorative Treatments	2 (1.2%)	97.1% (93.3, 99.0)	0.27 (−0.17, 0.71)
No Treatment	2 (1.2%)	96.5% (92.5, 98.7)	−0.02 (−0.03, − 0.003)
Bonding Agents	1 (0.6%)	95.9% (91.8, 98.3)	−0.01 (−0.03, 0.01)
Lasers	1 (0.6%)	99.4% (96.8, 100.0)	1.0 (1.0, 1.0)
Oxalates	1 (0.6%)	97.1% (93.3, 99.0)	−0.01 (−0.02, 0.01)
Sealants	0 (0%)	99.4% (96.8, 100.0)	---

*N* = 171

95% confidence intervals in parentheses

--- kappa not calculable due to the contingency table having a row containing only zero counts

Fluoride products were selected on the questionnaire by 62 dentists (36.3%) as their first choice. Among these, 33 (53.2%) indicated fluoride products most often in the clinical examinations. Overall agreement for the recommendation of fluoride products was 63.2% (95% CI: 55.5, 70.4) and kappa = 0.22 (95% CI: 0.07, 0.37).

The other recommendation options were selected as their first choice by 0 to 6 dentists (0 to 3.5%), which yielded relatively high percent agreement with observed rates for non-selection of these options. The lower 95% confidence limit for kappa includes zero for all but one recommendation, indicating no agreement beyond what would be expected by chance alone. The single exception was for use of lasers, which showed kappa = 1.0 (95% CI: 1.0, 1.0), based on only one dentist selecting this as the first-choice recommendation on the questionnaire. For this single dentist, laser treatment was also the most-used recommendation in the clinical data. There was complete agreement between the questionnaire and clinical observation for all other dentists regarding lasers not being their first choice of treatment. No dentists selected sealants as the first-choice product/recommendation, although for a single dentist this was the most commonly-indicated recommendation in the patient examinations.

Estimates of agreement with clinical recommendations for dentists’ indication of products/recommendations as being first, second or third choice options are shown in Table 2. Overall agreement was somewhat higher for indication of products/recommendations among the top three options. Fluoride products were selected on the questionnaire by 156 dentists (91.2%). Overall agreement with clinical recommendation of this product was 72.5% (95% CI: 65.2, 79.1), but kappa was only 0.08 (− 0.06, 0.22), indicating that agreement did not exceed that expected by chance.

**Table 2:** Agreement on treatment recommendation listed in top three choices for management of dentin hypersensitivity

Treatment Recommended	Product Listed in Top Three on Survey	Agreement Between Survey and Exam	Kappa
	n (%)	% (95% CI)	(95% CI)
Fluoride Products	156 (91.2%)	72.5% (65.2, 79.1)	0.08 (−0.06, 0.22)
Desensitizing OTC Potassium Nitrate Toothpaste	150 (87.7%)	73.1% (65.8, 79.6)	0.26 (0.12, 0.41)
Restorative Treatments	54 (31.6%)	70.8% (63.3, 77.5)	0.21 (0.07, 0.36)
Glutaraldehyde/HEMA Products	41 (24.0%)	77.8% (70.8, 83.8)	0.19 (0.04, 0.34)
Bonding Agents	34 (19.9%)	81.3% (74.6, 86.8)	0.29 (0.11, 0.47)
Advice	26 (15.2%)	60.8% (53.1, 68.2)	0.00 (−0.13, 0.13)
Other	16 (9.4%)	69.6% (62.1, 76.4)	0.22 (0.09, 0.34)
Oxalates	8 (4.7%)	91.8% (86.6, 95.5)	0.18 (−0.08, 0.44)
Sealants	8 (4.7%)	95.3% (91.0, 98.0)	0.18 (−0.14, 0.51)
No Treatment	7 (4.1%)	90.6% (85.3, 94.6)	0.23 (−0.02, 0.48)
Lasers	2 (1.2%)	100% (97.9, 100)	1.0 (1.0, 1.0)

N = 171

95% confidence intervals in parentheses

Desensitizing OTC potassium nitrate toothpaste was selected by 150 dentists (87.7%), and overall agreement with clinical recommendations was 73.1% (95% CI: 65.8, 79.6), kappa = 0.26 (95% CI: 0.12, 0.41).

Glutaraldehyde/HEMA products, bonding agents, restorative treatments, and advice were indicated as top-three choices for at least 15% of dentists, and showed overall agreement ranging from 60.8% (95% CI: 53.1, 68.2; kappa = 0.0) for advice to 81.3% (95% CI: 74.6, 86.8; kappa = 0.29) for bonding agents. Fewer than 10% of dentists selected any of the other options as among their first-, second- or third choices.

### Practitioner/practice characteristics and agreement

Only two practitioner characteristics showed statistically significant associations with agreement. Agreement on the recommendation of OTC potassium nitrate toothpaste differed by number of practice locations, with 76.2% agreement for practices having a single location, and 54.6% for practices with multiple locations (*p* = 0.0322). Agreement on the selection of advice among the top three recommendations differed by type of practice location. Inner city, urban (not inner city) suburban and rural locations showed 53.3, 38.3, 72.1 and 69.6% agreement, respectively (*p* = 0.0013). Results for all practitioner/practice characteristics are presented in Table 3 Appendix online [15].

## Discussion

The network provides an opportune setting in which to evaluate concordance between dental practitioners’ reported treatment preferences and methods and treatment choices as observed in an actual clinical setting. Analysis of concordance is necessary in order to establish the validity of questionnaire data to describe typical clinical practice. Data from the MDH study provides a context for the evaluation of this for a specific clinical scenario, that of DH, and was chosen as the basis for the current study because the MDH study collected both questionnaire and clinical data and so provided the opportunity to evaluate concordance. All of the patients who were enrolled in the MDH study had been diagnosed with DH, so neither incidence nor prevalence of DH is estimable from that study. These measures are not necessary to the evaluation of agreement between the two modes of collection of information.

Fluoride products and desensitizing OTC potassium nitrate toothpaste were the items selected by the most dentists as first-choice and as first-, second- or third-choice recommendations for management of DH. However, agreement between pre-study hypothetical questionnaire responses with observed rankings of items recommended during actual clinical examinations was modest for these items. Relatively high unadjusted agreement, often above 90%, was observed for some items selected by only a small number of dentists. This seems to be largely the result of dentists who did not favor the use of these items on the questionnaire also not tending to use them in clinical examinations.

Guidelines for characterization of agreement based on ranges of values of Cohen’s kappa statistic are arbitrary, but are often presented. A commonly-used set of such ranges [16], suggests that kappa greater than 0.75 indicates excellent agreement, 0.40 to 0.75 as fair to good, and below 0.40 as poor. Based on this, agreement on all items as ranked choices evaluated in this study was poor, with the exception of that for lasers. Only one dentist indicated lasers as first-choice, and two dentists indicated lasers as in the top three choices, and agreement between the survey responses and clinical recommendations was almost complete, at 99.4 and 100.0%, respectively. The survey did not address the availability of lasers in the practices.

The practitioners’ responses to the pre-study questionnaire are presumably based on their impressions of their typical practice. Characteristics of the patients who were enrolled in the clinical study might have differed from this expectation, and this could have affected agreement. In particular, practitioners who have multiple practice locations might consider different patient groups when completing the questionnaire, but clinical data were collected at only a single practice, so patient characteristics might differ from those of the aggregate.

It is not surprising that indication of a product/treatment as being in the top three would show higher agreement than that for first-choice. The top-three category is a larger target than the first-choice alone, and there are three chances for inclusion of a particular treatment recommendation. It is also possible that patients might have previously received a dentist’s first-choice recommendation without success, so that the dentist recommends a second- or third-choice approach for this patient.

A high percentage agreement for products/recommendations that were indicated by only a few dentists primarily reflects good agreement for non-use of those items. Dentists who did not choose these items on the pre-study questionnaire also did not tend to recommend them in the patient examinations.

Formal comparisons among agreement estimates in different studies may not be possible because of differences in methodology across studies. Previous network studies have reported concordance between dentists’ questionnaire responses and actual treatment practices regarding the choice of whether to repair or to replace dental restorations [3], between self-reported treatment planning thresholds and clinical choices [4], between stated treatment thresholds and clinical pre-treatment depth estimates [2] and between pre-operative and post-operative estimates of caries lesion depths [1]. Nonetheless, comparisons with other network studies may be of interest. Agreement for the two most-frequent recommendations in the current study were 63.7% for OTC potassium nitrate toothpaste and 63.2% for fluoride products.

Higher agreement levels might be expected for pre- and post-treatment clinical observations of the same clinical entity, such as the same caries lesion. For example, in one network study that quantified the agreement between pre- and post-intervention estimates of caries lesion depth, agreement of 88% was observed for lesions extending to the inner one-third of dentin and 54% for lesions limited to the outer one-half of enamel [1]. However, excellent agreement has also been found between stated treatment thresholds and pre-treatment estimates of caries lesion depth for lesions that were on the proximal surface (98% agreement), but agreement for occlusal lesions was only 51% in the same study [2].

## Conclusions

Agreement percentages between hypothetical, typical clinical scenarios (based on questionnaire data) and actual practice (based on clinical examination data) were generally high, but low values of Cohen’s kappa indicated little agreement beyond levels that would be expected by chance. Based on this, survey data and hypothetical scenarios could supplement observations based on actual examinations in studies of DH treatment recommendations, but these should not take the place of data based on clinical observations. This analysis adds to the larger work of the network which has observed a wide range of levels of agreement between hypothetical and actual care, depending upon the specific diagnosis or treatment under consideration.

### Additional files

Additional file 1: Sensitive Teeth Study Online Practitioner Questionnaire. Pre-Study Questionnaire: Information on practitioners’ diagnosis and treatment modalities used when seeing patients presenting with a dentin hypersensitivity complaint. (PDF 226 kb)

Additional file 2: Sensitive Teeth Study Baseline Exam. Patient Baseline Examination Form: Information on number and location of sensitive teeth and recommended treatments. (PDF 221 kb)

Additional file 3: STROBE Checklist. Completed STROBE Checklist (DOC 79 kb)

## Appendix

**Table 3:** Association of practitioner and practice characteristics with agreement on the practitioners’ top three treatment recommendations

Treatment Recommendation	Practitioner Characteristic	Categories	Agreement (%)	*p*-value
Fluoride	Age Group	27–39	69.7	0.9212
		40–49	70.6	
		50–59	72.4	
		60+	76.1	
	Gender	Male	71.7	0.7601
		Female	73.9	
	Race	White	71.9	0.7742
		Other	74.3	
	Full Time/Part Time	Full	72.3	0.9964
		Part	72.7	
	Practice Location Type	Inner city	80.0	0.9017
		Urban (not inner city)	70.2	
		Suburban	72.1	
		Rural	73.9	
	Number of Locations	1	73.5	0.6033
		2+	68.2	
OTC Potassium nitrate toothpaste	Age Group	27–39	87.9	0.1647
		40–49	64.7	
		50–59	70.7	
		60+	71.7	
	Gender	Male	74.5	0.5905
		Female	70.8	
	Race	White	72.6	0.8408
		Other	74.3	
	Full Time/Part Time	Full	72.3	0.6240
		Part	77.3	
	Practice Location Type	Inner city	86.7	0.2274
		Urban (not inner city)	68.1	
		Suburban	76.7	
		Rural	60.9	
	Number of Locations	1	76.2	0.0322
		2+	54.6	
Glutaraldehyde / HEMA	Age Group	27–39	63.6	0.0805
		40–49	73.5	
		50–59	86.2	
		60+	80.4	
	Gender	Male	75.5	0.3543
		Female	81.5	
	Race	White	80.0	0.1481
		Other	68.6	
	Full Time/Part Time	Full	77.7	0.9640
		Part	77.3	
	Practice Location Type	Inner city	60.0	0.1722
		Urban (not inner city)	72.3	
		Suburban	82.6	
		Rural	82.6	
	Number of Locations	1	78.2	0.9192
		2+	77.3	
Bonding Agents	Age Group	27–39	81.8	0.6491
		40–49	76.5	
		50–59	79.3	
		60+	87.0	
	Gender	Male	79.3	0.3821
		Female	84.6	
	Race			
		White	80.0	0.4409
		Other	85.7	
	Full Time/Part Time	Full	80.4	0.5047
		Part	86.4	
	Practice Location Type	Inner city	93.3	0.0776
		Urban (not inner city)	70.2	
		Suburban	82.6	
		Rural	91.3	
	Number of Locations	1	81.6	0.9833
		2+	81.8	
Sealants	Age Group	27–39	21.2	0.3074
		40–49	35.3	
		50–59	36.2	
		60+	41.3	
	Gender	Male	34.0	0.8494
		Female	35.4	
	Race	White	33.3	0.4603
		Other	40.0	
	Full Time/Part Time	Full	33.8	0.5124
		Part	40.9	
	Practice Location Type	Inner city	40.0	0.4393
		Urban (not inner city)	34.0	
		Suburban	30.2	
		Rural	47.8	
	Number of Locations	1	33.3	0.4852
		2+	40.9	
Restorative Treatments	Age Group	27–39	75.8	0.6353
		40–49	15.2	
		50–59	69.0	
		60+	65.2	
	Gender	Male	69.8	0.7276
		Female	72.3	
	Race	White	69.6	0.5901
		Other	74.3	
	Full Time/Part Time	Full	68.9	0.0919
		Part	86.4	
	Practice Location Type	Inner city	73.3	0.7303
		Urban (not inner city)	76.6	
		Suburban	67.4	
		Rural	69.6	
	Number of Locations	1	68.7	0.2089
		2+	81.8	
Oxalates	Age Group	27–39	90.9	0.9588
		40–49	94.1	
		50–59	91.4	
		60+	91.3	
	Gender	Male	92.5	0.6967
		Female	90.8	
	Race	White	90.4	0.1940
		Other	97.1	
	Full Time/Part Time	Full	93.2	0.0689
		Part	81.8	
	Practice Location Type	Inner city	100.0	0.2253
		Urban (not inner city)	89.4	
		Suburban	89.5	
		Rural	100.0	
	Number of Locations	1	91.8	0.8830
		2+	90.9	
No Treatment	Age Group	27–39	90.9	0.3070
		40–49	82.4	
		50–59	93.1	
		60+	93.5	
	Gender	Male	89.6	0.5584
		Female	92.3	
	Race	White	90.4	0.8485
		Other	91.4	
	Full Time/Part Time	Full	90.5	0.9559
		Part	90.9	
	Practice Location Type	Inner city	80.0	0.4435
		Urban (not inner city)	89.4	
		Suburban	93.0	
		Rural	91.3	
	Number of Locations	1	91.8	0.1344
		2+	81.8	
Advice	Age Group	27–39	72.7	0.3679
		40–49	55.9	
		50–59	62.1	
		60+	54.4	
	Gender	Male	65.1	0.1436
		Female	53.9	
	Race	White	60.7	0.9363
		Other	60.0	
	Full Time/Part Time	Full	60.8	0.8776
		Part	59.1	
	Practice Location Type	Inner city	53.3	0.0013
		Urban (not inner city)	38.3	
		Suburban	72.1	
		Rural	69.6	
	Number of Locations	1	61.2	0.8483
		2+	59.1	
Other	Age Group	27–39	69.7	0.3813
		40–49	64.7	
		50–59	77.6	
		60+	63.0	
	Gender	Male	68.9	0.7930
		Female	70.8	
	Race	White	68.2	0.4825
		Other	74.3	
	Full Time/Part Time	Full	70.3	0.5286
		Part	63.6	
	Practice Location Type	Inner city	60.0	0.5872
		Urban (not inner city)	76.6	
		Suburban	67.4	
		Rural	69.6	
	Number of Locations	1	67.4	0.0699
		2+	86.4	

## Abbreviations

CI: Confidence interval
DH: Dentin hypersensitivity
MDH: Management of dentin hypersensitivity
