# Supplementary material for: Concordance between practitioner questionnaire responses and observed clinical treatment recommendations for treatment of dentin hypersensitivity: findings from the National Dental Practice-Based Research Network
Source: BMC Oral Health. 2019 Jun 14;19:112. doi: 10.1186/s12903-019-0772-y (PMC6570951; doi:10.1186/s12903-019-0772-y)
Supplement: Supplementary file 1 — Sensitive Teeth Study Online Practitioner Questionnaire. Pre-Study Questionnaire: Information on practitioners’ diagnosis and treatment modalities used when seeing patients presenting with a dentin hypersensitivity complaint. (PDF 226 kb) [file 12903_2019_772_MOESM1_ESM.pdf]

SENSITIVE TEETH STUDY
ONLINE PRACTITIONER QUESTIONNAIRE

Dear Colleague:

We are pleased you have decided to participate in the Management of Dentin Hypersensitivity (MDH) study sponsored through the National Dental Practice-Based Research Network (PBRN).

Your next step is the completion of this questionnaire to assess the National Dental PBRN practitioners' diagnosis and treatment modalities used when seeing patients that present with a dentin hypersensitivity complaint. **We estimate that completing this questionnaire will take up to 10 minutes for which you or your practice organization will receive a $50 payment.**

Your participation and responses will remain confidential. Only authorized study personnel will have access to data. All information will be stored in a secure manner. **Your information will not be sold, used for any reason other than research, released to any insurance company, or released to any other similar interest.** Information from this questionnaire along with information from other network studies you have completed or will complete, may be linked using your assigned practitioner ID number. This will allow us to see how characteristics from each study might be related to each other.

Results may be published for scientific purposes, but your identity will not be revealed. Only statistical summaries will be presented. The University of Alabama at Birmingham (UAB) Institutional Review Board (IRB) maintains the authority to inspect completed questionnaires to ensure compliance with IRB procedures.

If you have any additional questions, please contact your Regional Coordinator [LINK to Public Website].

With regards,

Dorota T. Kopycka-Kedzierawski DDS, MPH
Study Principal Investigator

SECTION 1: ELIGIBILITY

1. Do you see patients 19 years of age and older in your practice?

☐ Yes
☐ No

SECTION 2: DIAGNOSIS OF DENTIN HYPERSENSITIVITY

This section concerns how you diagnose dentin hypersensitivity.

2. How do you routinely *diagnose* dentin hypersensitivity? **Please check all that apply:**

☐ Spontaneous patient report confirmed by the dental exam
☐ Patient report after dentist's query
☐ Applying air blast
☐ Applying cold water
☐ Scratching dentin with dental explorer
☐ Requesting numeric rating of pain
☐ Requesting visual analog scale rating of pain
☐ Using electric pulp tester
☐ Other (please specify):____________________________________

3. Which single method do you use most frequently to *diagnose* dentin hypersensitivity? **Please check a single answer:**

☐ Spontaneous patient report confirmed by the dental exam
☐ Patient report after dentist's query
☐ Applying air blast
☐ Applying cold water
☐ Scratching dentin with dental explorer
☐ Requesting numeric rating of pain
☐ Requesting visual analog scale rating of pain
☐ Using electric pulp tester
☐ Other (please specify):____________________________________

SECTION 3: MANAGEMENT/TREATMENT OF DENTIN HYPERSENSITIVITY

This section concerns how you manage and treat dentin hypersensitivity.

4. What treatment modalities do you routinely use to *manage* dentin hypersensitivity? **Please check all that apply:**

SAMPLE

☐ Fluoride products (e.g., gels, varnishes, pastes, rinses)
☐ Desensitizing OTC potassium nitrate toothpastes
☐ Glutaraldehyde/HEMA products
☐ Bonding agents
☐ Sealants
☐ Restorative treatments
☐ Lasers
☐ Oxalates
☐ No treatment
☐ Advice (please specify):_____________________________________
☐ Other (please specify):______________________________________

5. Please indicate in the table below your **first, second and third <u>choice</u>** of products to prescribe/recommend to your patients when managing dentin hypersensitivity?

Note: Online this question will be displayed as 5a and 5b – 5a will display all choices selected in question 4 and ask for the top three choices; Question 5b will ask for the first, second, and third choice.

Product	First Choice	Second Choice	Third Choice
Fluoride products (e.g., gels, varnishes, pastes, rinses)	☐	☐	☐
Desensitizing OTC potassium nitrate toothpastes	☐	☐	☐
Glutaraldehyde/HEMA products	☐	☐	☐
Bonding agents	☐	☐	☐
Sealants	☐	☐	☐
Restorative treatments	☐	☐	☐
Lasers	☐	☐	☐
Oxalates	☐	☐	☐
No treatment	☐	☐	☐
Advice (please specify):______________________________	☐	☐	☐
Other:<br>(please specify):____________________________________	☐	☐	☐

6. Do you recommend <u>combinations</u> of products when managing dentin hypersensitivity?
☐ Yes
☐ No (**SKIP TO Q8**)

SAMPLE

7. What is the combination of products you recommend <u>most often</u>?

<u>Please check all that apply</u>:

- ☐ Fluoride varnish and desensitizing OTC potassium nitrate toothpaste
- ☐ Glutaraldehyde/HEMA products and desensitizing OTC potassium nitrate toothpaste
- ☐ Bonding agents and desensitizing OTC potassium nitrate toothpaste
- ☐ Glutaraldehyde/HEMA products and fluoride prescription toothpaste
- ☐ Sealants and desensitizing OTC potassium nitrate toothpaste
- ☐ Restorative treatments and desensitizing OTC potassium nitrate toothpaste
- ☐ Other (please specify):____

<u>SECTION 4: PREDISPOSING FACTORS OF DENTIN HYPERSENSITIVITY</u>

This section concerns your thoughts on the predisposing factors of dentin hypersensitivity.

8. In your opinion, which of the following factors most likely predispose a patient to dentin hypersensitivity? **<u>Please check all that apply</u>:**

- ☐ Overbrushing/Brushing aggressively
- ☐ Excessive flossing
- ☐ Excessive teeth whitening
- ☐ Extensive periodontal surgery
- ☐ Scaling and root planing
- ☐ Recessed gingiva
- ☐ Bruxism
- ☐ Smoking
- ☐ Abrasion, erosion, abfraction and/or attrition
- ☐ Frequent consumption of citrus juices, and/or carbonated drinks
- ☐ Gastric reflux and/or excessive vomiting
- ☐ Trauma during tooth preparation
- ☐ Anatomy of cementoenamel junction
- ☐ Other (please specify):_____________________________

9. Please indicate in the table below your **first, second and third <u>choice</u>** of factors that most likely predispose a patient to dentin hypersensitivity?

Note: Online this question will be displayed as 9a and 9b – 9a will display all choices selected in question 8 and ask for the top three choices; Question 9b will ask for the first, second, and third choice.

Predisposing factor	First Choice	Second Choice	Third Choice
Overbrushing/Brushing aggressively	☐	☐	☐

SAMPLE

Excessive flossing	☐	☐	☐
Excessive teeth whitening	☐	☐	☐
Extensive periodontal surgery	☐	☐	☐
Scaling and root planing	☐	☐	☐
Recessed gingiva	☐	☐	☐
Bruxism	☐	☐	☐
Smoking	☐	☐	☐
Abrasion, erosion, abfraction and/or attrition	☐	☐	☐
Frequent consumption of citrus juices, and/or carbonated drinks	☐	☐	☐
Gastric reflux and/or excessive vomiting	☐	☐	☐
Trauma during tooth preparation	☐	☐	☐
Anatomy of cementoenamel junction	☐	☐	☐
Other (please specify):___________________________________	☐	☐	☐

10. Is there anything else you think we should know about regarding how you diagnose and treat dentin hypersensitivity?

[OPEN FIELDS FOR TEXT ENTRY BY THE RESPONDENT HERE-250 characters]

11. Would you like us to send you or your practice organization $50.00 as a thank you for completing this questionnaire?

- ☐ Yes, please send compensation.
- ☐ No [GOTO END OF QUESTIONNAIRE]
- ☐ Excluded from payment.

[END OF QUESTIONNAIRE]

Thank you for your completing this questionnaire!

SAMPLE
