# Supplementary material for: Concordance between practitioner questionnaire responses and observed clinical treatment recommendations for treatment of dentin hypersensitivity: findings from the National Dental Practice-Based Research Network
Source: BMC Oral Health. 2019 Jun 14;19:112. doi: 10.1186/s12903-019-0772-y (PMC6570951; doi:10.1186/s12903-019-0772-y)
Supplement: Supplementary file 2 — Sensitive Teeth Study Baseline Exam. Patient Baseline Examination Form: Information on number and location of sensitive teeth and recommended treatments. (PDF 221 kb) [file 12903_2019_772_MOESM2_ESM.pdf]

Pre-printed SID number

ORANGE

The National Dental Practice-Based Research Network
The nation's network

SENSITIVE TEETH STUDY

Baseline Exam

Visit Date: |___|___|/|___|___| /|_2_|_0_||_1_|___|
m m d d y y y y

Dentist-assessed signs of dentin hypersensitivity

1. Fill in the boxes below: (1) Circle the number of each tooth/teeth judged sensitive in the upper arch. (2) Mark the restoration(s) present on the sensitive tooth/teeth only.

2	3	4	5	6	7	8	9	10	11	12	13	14	15	B/F
☐	☐	☐	☐	☐	☐	☐	☐	☐	☐	☐	☐	☐	☐	☐ L

2. Check: (1) Whether visible dentin and (2) gingival recession is present and (3) the type of the restoration only on the hypersensitive upper tooth/teeth chosen above. **Mark all that apply**:

	2	3	4	5	6	7	8	9	10	11	12	13	14	15
Visible Dentin	☐	☐	☐	☐	☐	☐	☐	☐	☐	☐	☐	☐	☐	☐
Gingival Recession	☐	☐	☐	☐	☐	☐	☐	☐	☐	☐	☐	☐	☐	☐
No Restoration	☐	☐	☐	☐	☐	☐	☐	☐	☐	☐	☐	☐	☐	☐
Amalgam	☐	☐	☐	☐	☐	☐	☐	☐	☐	☐	☐	☐	☐	☐
Resin	☐	☐	☐	☐	☐	☐	☐	☐	☐	☐	☐	☐	☐	☐
PFM Restoration	☐	☐	☐	☐	☐	☐	☐	☐	☐	☐	☐	☐	☐	☐
All Porcelain	☐	☐	☐	☐	☐	☐	☐	☐	☐	☐	☐	☐	☐	☐
Cast Metal	☐	☐	☐	☐	☐	☐	☐	☐	☐	☐	☐	☐	☐	☐
Other Restoration	☐	☐	☐	☐	☐	☐	☐	☐	☐	☐	☐	☐	☐	☐

SAMPLE

3. Fill in the boxes below: (1) Circle the number of each tooth/teeth judged sensitive in the lower arch. (2)Mark the restoration(s) present on the sensitive tooth/teeth only.

☐	☐	☐	☐	☐	☐	☐	☐	☐	☐	☐	☐	☐	☐
31	30	29	28	27	26	25	24	23	22	21	20	19	18

4. Check: (1) Whether visible dentin and (2) gingival recession is present and (3) the type of the restoration only on the hypersensitive lower tooth/teeth chosen above. **Mark all that apply**:

	31	30	29	28	27	26	25	24	23	22	21	20	19	18
Visible Dentin	☐	☐	☐	☐	☐	☐	☐	☐	☐	☐	☐	☐	☐	☐
Gingival Recession	☐	☐	☐	☐	☐	☐	☐	☐	☐	☐	☐	☐	☐	☐
No Restoration	☐	☐	☐	☐	☐	☐	☐	☐	☐	☐	☐	☐	☐	☐
Amalgam	☐	☐	☐	☐	☐	☐	☐	☐	☐	☐	☐	☐	☐	☐
Resin	☐	☐	☐	☐	☐	☐	☐	☐	☐	☐	☐	☐	☐	☐
PFM Restoration	☐	☐	☐	☐	☐	☐	☐	☐	☐	☐	☐	☐	☐	☐
All Porcelain	☐	☐	☐	☐	☐	☐	☐	☐	☐	☐	☐	☐	☐	☐
Cast Metal	☐	☐	☐	☐	☐	☐	☐	☐	☐	☐	☐	☐	☐	☐
Other Restoration	☐	☐	☐	☐	☐	☐	☐	☐	☐	☐	☐	☐	☐	☐

SAMPLE

5. Total number of natural teeth present: |___|___| (**3rd molars excluded**)

6. Total number of natural posterior teeth present: |___|___|

7. Patient out of pocket expense for the preventive visit and/or problem focused exam will be: **(Check one)**

☐ Both visit types will be covered 100% by the insurance
☐ Patient will pay a co-payment for both visit types
☐ Patient will pay 100% out of the pocket for both visit types

Pre-printed SID number

ORANGE

8. Treatment recommended and/or prescribed: (**Check and complete all that apply**)

	Treatment recommended and/or prescribed	Duration	Frequency
☐	Fluoride (if checked, specify duration and frequency):		
☐	Fluoride Gel (if checked, specify duration and frequency):		
☐	Fluoride Varnish (if checked, specify duration and frequency):		
☐	Fluoride Paste (if checked, specify duration and frequency):		
☐	Fluoride Rinse (if checked, specify duration and frequency):		
☐	Desensitizing OTC Potassium nitrate toothpastes (if checked, specify duration and frequency):		
☐	Glutaraldehyde/HEMA products		
☐	Bonding agents		
☐	Sealants		
☐	Restorative treatments		
☐	Lasers		
☐	Oxalates		
☐	No treatment		
☐	Advice (If checked, specify below. Check all that apply):<br>☐ To stop product, habit and/or behavior. If yes, what product/habit/behavior?<br>_______________<br>☐ To decrease product, habit and/or behavior. If yes, what product/habit/behavior?<br>_______________<br>☐ To start or increase behavior. If yes, what behavior?<br>_______________		
☐	Other (please specify):_______________		

9. Recommended follow-up time for this patient's sensitive tooth/teeth: **(Check one)**

☐ More than 4 weeks
☐ 2-4 weeks
☐ 2 weeks
☐ No follow-up is needed
☐ Other, (Please specify :) ____________________

____
Practitioner Signature

Date: |___|___|/|___|___| /|_2_|_0_|_1_|___|
m m d d y y y y

PLEASE REVIEW THIS FORM FOR COMPLETION AND ACCURACY. THANK YOU.

Questions? Contact your Regional Coordinator.

SAMPLE
